# Financial Incentives for Adherence to Hepatitis C Virus Clinical Care and Treatment: A Randomized Trial of Two Strategies

**DOI:** 10.1093/ofid/ofx095

**Published:** 2017-05-05

**Authors:** David A. Wohl, Andrew G. Allmon, Donna Evon, Christopher Hurt, Sarah Ailleen Reifeis, Harsha Thirumurthy, Becky Straub, Angela Edwards, Katie R. Mollan

**Affiliations:** 1 Institute of Global Health and Infectious Diseases, The University of North Carolina at Chapel Hill; 2 The University of North Carolina Center for AIDS Research and the Department of Biostatistics, Gillings School of Global Public Health, The University of North Carolina at Chapel Hill; 3 Division of Gastroenterology and Hepatology, The University of North Carolina at Chapel Hill; 4 The University of North Carolina Center for AIDS Research and the University of North Carolina Lineberger Cancer Center; and; 5 Department of Health Policy and Management, Gillings School of Global Public Health, The University of North Carolina at Chapel Hill

**Keywords:** adherence, direct-acting agents, financial incentives, HCV, substance use

## Abstract

**Background:**

Although rates of sustained virologic response (SVR) after hepatitis C virus (HCV) treatment with direct-acting antivirals (DAAs) surpass 90% in trials and some more “real world” settings, some patients, such as those with substance use disorders, will be challenged to adhere to HCV care.

**Methods:**

To assess the feasibility of 2 strategies for financially incentivizing adherence to HCV care, patients with a substance use history prescribed 12 weeks of a sofosbuvir-containing regimen were randomized to either fixed or lottery-based monetary incentives for attending clinic appointments, pill count adherence >90%, and SVR achievement. Electronic medication monitoring provided an objective measure of DAA adherence.

**Results:**

Fifty-nine participants were randomized to the lottery (n = 31) or fixed-incentive (n = 28) arms. All 31 (100%) in the lottery arm and 24 of 28 (86%) in the fixed arm completed 12 weeks of therapy. By intent-to-treat, 93% in the lottery arm and 92% in the fixed arm achieved SVR (estimated difference: 0.5%; 95% confidence interval, −17.5 to 18.8). Overall, 92% of scheduled visits were attended without significant differences between arms. The mean adherence ratio (days with ≥1 bottle opening:monitored days) was 0.91 for lottery and 0.92 for fixed arms.

**Conclusions:**

In this pilot, fixed- and lottery-based financial incentives were successfully implemented and accepted by patients with a substance use history. High levels of HCV therapy and care adherence, as well as rates of SVR, were observed. Financial incentives may be useful to support treatment adherence in patients with substance use disorders and should be tested in a larger, randomized, controlled trial.

Direct-acting antivirals (DAAs) have revolutionized the management of hepatitis C virus (HCV) infection, the most prevalent chronic bloodborne pathogen in the United States. With sustained virologic response (SVR) rates of 85%–95%, these medications are potent but also very well tolerated and convenient, and the usual course of treatment is often 12 weeks [1–6].

In clinical trials and observational studies, failure to respond to DAA regimens has been ascribed to a number of factors including drug-drug interactions, host genetics, and inherent viral drug resistance [7, 8]. However, medication adherence has also been implicated in suboptimal HCV treatment outcomes, and, as the number of real-world patients who receive treatment increases, there are concerns that rates of virologic response will be lower than those observed in clinical research studies [9–15]. In particular, there are questions regarding the ability of those with substance abuse disorders to adhere to a full-course HCV therapy, given the social instability and mental health challenges that frequently characterize the lives of these individuals [14–16].

Studies conducted in the pre-DAA era examining an association between substance use and HCV treatment outcomes such as adherence, treatment discontinuation, and virologic response have produced mixed results [16]. Data regarding adherence to the newest DAAs are sparse. Pill count and electronic medication assessments during DAA clinical trials have found high adherence levels, even among the few participants with active substance use or who were receiving opioid substitution therapy [17–19]. To participate in clinical research studies, patients must meet criteria that are designed to exclude those at the highest risk of nonadherence, including heavy users of illicit substances. In one analysis, less than 10% of patients living with HCV infection in Canada would meet eligibility criteria of 4 of the 5 major clinical trials that, by the end of 2015, had led to second-generation DAA approval, with most patients excluded due to active illicit substance use [20].

Observational studies examining prescription refills among HCV-infected patients receiving community care also demonstrate high rates of adherence to newer DAA regimens [21, 22]. Sustained virologic response rates approximating those reported from clinical trials have also been observed in such studies [21–23]. However, although such “real-world” experiences are encouraging, these studies are also not without potential selection bias. Clinicians often are restrictive in their criteria for treatment eligibility, and many continue to deem patients with active substance use and mental health disorders as poor candidates for HCV therapy. Moreover, some state Medicaid programs make approval of HCV therapy coverage contingent upon months of documentation abstinence. An analysis of HCV treatment outcome data from over 4000 patients in the Veterans Administration system, which generally has less restrictive criteria for HCV therapy, found SVR rates that were lower than those seen in clinical trials [11].

Expanding HCV therapy to “all-comers” will lead to the treatment of a greater number of patients who may have difficulty adhering to medication and clinic visits [14–16]. Although the proportion of the estimated 4 million HCV-infected persons in the United States who may be suboptimally adherent to a DAA regimen is relatively small, the absolute number of such patients and the costs of treatment failure are not. At present, there is a limited evidence base on interventions that can support adherence to these medications, especially for individuals challenged with chronic or active substance use disorders [24], and although there have been calls by some HCV care providers for the integration of HCV care within primary care clinics and addiction treatment centers, models of such programs remain untested in controlled studies [25, 26].

In contrast, an approach that has been demonstrated in randomized, controlled trials to positively influence health behaviors is financial incentivization [27–39]. Financial incentives using various strategies have been applied to promote weight loss, smoking cessation, tuberculosis treatment, and antiretroviral therapy [27–30]. Such incentivization may be effective not only through offsetting the cost of clinic attendance for patients but also by offering immediate rewards for behaviors that otherwise have a delayed benefit [37]. In particular, financial incentive interventions have proven especially potent for achieving and maintaining sobriety and abstinence from substance use [30–39].

In this pilot study, we explored the feasibility of implementing financial incentives for DAA and clinic visit adherence among HCV-infected men and women with a history of substance use. Two commonly used strategies—fixed versus lottery-based incentives—on adherence to DAA adherence, routine HCV clinical care visits, and achievement of SVR were studied.

## METHODS

### Study Design and Participants

Participants in this single-center, randomized clinical trial were individuals age 18 years or greater with documented chronic HCV infection (HCV seropositive and detectable plasma HCV ribonucleic acid [RNA]) who were prescribed a 12-week course of a sofosbuvir-based HCV therapy regimen. At the time the study was developed and launched, sofosbuvir was the most commonly prescribed second-generation DAA approved for use in the United States. All participants had to self-report a history of substance use for at least 1 year in their lifetime, be English-speaking, and be able and willing to provide informed consent. All participants were patients of either the University of North Carolina (UNC) Infectious Diseases Clinic or Liver Center. Data were collected during routine clinic visits for HCV care. Hepatitis C virus treatment was selected by the participant’s clinician before study referral and entry/randomization. The research protocol, consent documents, and recruitment materials were approved by the UNC Institutional Review Board.

### Study Arms

After consent, participants were randomized 1:1 to 1 of 2 study arms: either fixed or lottery-based financial incentives to reinforce clinic attendance and medication adherence. The incentive amount was chosen to offset clinic transportation costs as well as lost wages and also to appear large enough to motivate health-seeking behavior. Those in the fixed-incentive arm received $40 for each scheduled HCV-related clinic appointment attended (or if rescheduled within 5 business days of their original appointment) and $20 for HCV medication pill count adherence of >90%. A bonus of $50 was provided for achievement of an undetectable HCV viral load at the end of treatment (EOT) and at 12 weeks postcompletion of treatment (ie, SVR). Incentives provided in the lottery-based incentive arm occurred on the same schedule as fixed incentives and for the same milestones. However, participants in this arm were asked to draw a card from a bag to determine the amount they received for each milestone. Cards were printed with one of the following values: $10, $30, and $100. Participants who qualified for a clinic attendance lottery draw chose 1 card from a bag that contained cards with the following probabilities: 20% for $100, 30% for $30, and 50% for $10. Participants who qualified for a pill count adherence lottery draw chose 1 card from a bag that contained cards with the following probabilities: 10% for $50, 40% for $20, and 50% for $10. A bonus of 2 lottery draws from the clinic attendance bag was provided for achievement of an undetectable HCV viral load at EOT and SVR. For both study arms, all financial incentives were provided in the form of cash and paid immediately, except for the incentive based on the achievement of SVR, which was paid after return of the determining HCV RNA result.

### Assessments and Outcomes

After the baseline visit, study visits were conducted on the same day that participants came for their routinely scheduled HCV-related clinic appointments. These routine visits generally were scheduled monthly during the 12 weeks of active HCV therapy and then at EOT and 12 weeks posttreatment.

#### Surveys and Record Review

At baseline, demographic information, medical history, self-reported alcohol and substance use (Alcohol Use Disorders Identification Test [AUDIT] [40], Drug Abuse Screen Test [DAST] [41], Texas Christian University Drug Screen-II [42]), and depression screening (Center for Epidemiologic Studies Depression Scale [CES-D] [43]) responses were collected by a research assistant via computer-assisted participant interviewing (CAPI) using a web-based survey platform (Qualtrics, Provo, UT). At subsequent visits, alcohol, substance use, and depression surveys were administered. Medical records were reviewed to confirm clinic visit attendance and laboratory results. At the last study visit, semistructured interviews were conducted among 15 participants to obtain their perspectives regarding HCV treatment and the financial incentive strategy to which they were assigned.

#### Medication Adherence

Adherence to HCV medications was assessed using electronic medication monitoring (MEMSCap; WestRock Switzerland, Ltd) and pill count. For each participant, an electronic monitoring device was placed on her/his sofosbuvir-containing medication bottle. The cap recorded the time and date of each bottle opening, and these data were downloaded at each visit during the treatment course. Participants were asked at each visit about use of the electronic cap, including diversion of pills from the monitored bottle to other containers or pill boxes. Cap opening data were used as a measure of medication adherence and were not shared with the participant, nor were cap data used to trigger financial incentives. For pill counts, the number of tablets in each bottle of oral HCV medication were counted and the proportion of tablets present was compared with the proportion expected. The resultant ratio was categorized as <90% (suboptimal adherence), 90%–100% (optimal adherence), >100% (over adherence).

#### Plasma Hepatits C Virus Ribonucleic Acid

Quantitative HCV RNA measurements were conducted according to standard of care as determined by the participants’ clinicians. Testing was conducted at the UNC McLendon Clinical Laboratories or commercial clinical laboratories, and results were abstracted from electronic medical records. An HCV RNA level obtained >8 weeks after completion of therapy was used to determine achievement of SVR.

#### Urine Toxicology Screening

At baseline and subsequent visits, participants were asked to provide urine to screen for the presence of cocaine, opiates, benzodiazepines, and amphetamines not expected to be present based on self-reported and medical record medication lists.

### Statistical Methods

The primary aim of this study was to assess the feasibility of fixed versus lottery-based financial incentives to improve adherence to DAAs and to attendance at treatment-related visits among HCV-infected patients with a substance use history. Feasibility was assessed by describing successful implementation of the interventions, study retention rates, and the completion of study assessments.

Medication adherence was assessed using pill count and electronic cap data as a secondary outcome. For electronic cap data, when the number of bottle openings matched or exceeded the expected dose frequency, adherence for the day was considered a success. An adherence ratio was calculated as the number of successful adherence days divided by the number of monitored days; HCV DAAs were prescribed to be taken once daily. The overall proportion of participants with an electronic cap adherence level ≥90% and the difference in proportions between arms was estimated with a corresponding exact Chan and Zhang [44] 95% confidence interval (CI). Electronic cap data were analyzed using 2 approaches: (1) a conservative analysis in which no adjustment to the recorded data were made and (2) an adjudicated analysis in which the electronic cap data were censored based on participant self-report of nonuse of the cap.

An additional secondary aim of this study was to determine the proportion of participants who achieved SVR. An intent-to-treat (ITT) analysis was complete-case and included all participants who were randomized and who had an HCV RNA level obtained >8 weeks after the treatment regimen ended. A per-protocol (PP) analysis was also conducted and included participants in the ITT analysis sample who completed a 12-week regimen, but unlike the ITT analysis it excluded participants (n = 5) whose treatment durations were modified during the study to either an 8- or 24-week course. The overall proportion of participants who achieved SVR was estimated with a corresponding exact Clopper-Pearson 95% CI [45]. The proportion of participants in each study arm who achieved SVR was calculated, and the difference in proportions between arms was estimated with a corresponding exact Chan and Zhang [44] 95% CI.

The effect of substance use on the outcomes of interest was examined by the following: (1) estimating the proportion ratio (PR) for achievement of SVR among participants whose last on-study urine samples tested positive for substances of abuse versus those participants who tested negative for these substances and (2) estimating the PR for achievement of an adjudicated adherence level of ≥90% for those whose baseline urine sample tested positive for substances of abuse versus those who tested negative. The last urine toxicology screen was used in the SVR analysis to represent substance use during the study period while minimizing missing data, and the baseline urine screen was used in the adherence analysis to measure substance use near the start of HCV treatment. Participants were excluded from these analyses if they were missing the relevant urine screen data, final SVR status, or adherence status. The PR was estimated with a corresponding exact Chan and Zhang [44] 95% CI. All statistical analyses were conducted in SAS version 9.4 (SAS Institute, Cary, NC) or StatXact-11 (Cytel, Inc., Cambridge, MA), and a 2-sided 0.05 significance level was used for statistical inference.

## RESULTS

### Study Participants

**Figure 1.**
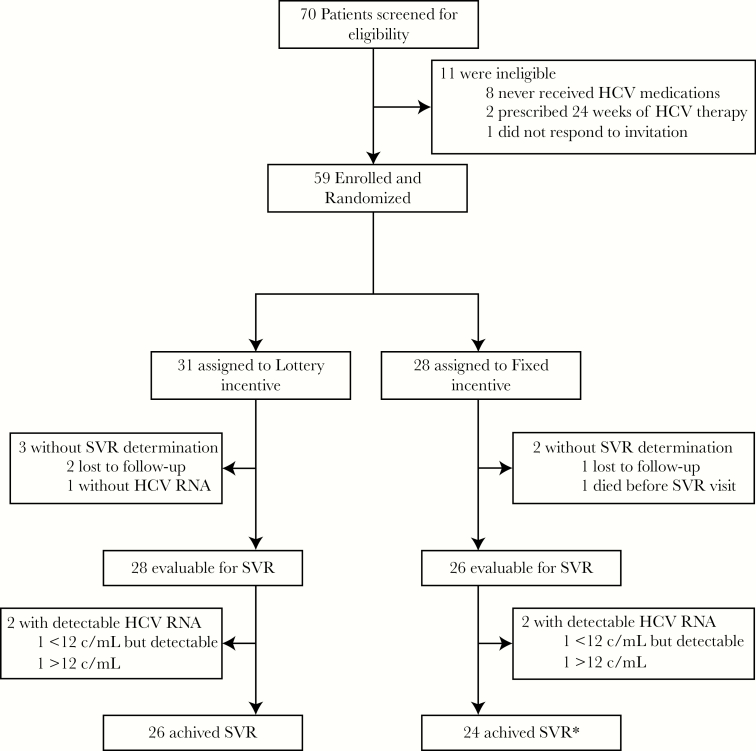
Screening, randomization, and follow-up analyses. HCV, hepatitis C virus; RNA, ribonucleic acid; SVR, sustained virologic response.

Seventy patients were consented, screened, and entered into the trial (Figure 1). Of these, 11 were not enrolled, mostly due to an inability to receive payer approval for coverage of their prescribed DAA regimen. The remaining 59 participants were randomized to the lottery-based (n = 31) or fixed incentive (n = 28) study arms. The majority (68%) of the participants were men and the median age was 54 years; half were nonwhite (Table 1). One third (32%) were known to be human immunodeficiency virus-coinfected, and 41% had a diagnosis of cirrhosis documented in the medical record. At baseline, 46% reported that they were actively using illicit substances. The CES-D scores indicated that clinical depression was prevalent, with 71% scoring above 16, a threshold used to indicate presence of clinical depression. Six (10%) of the particiants were classified at baseline as engaging in hazardous drinking based on an AUDIT score of 8 or greater. Hepatitis C virus genotype 1 accounted for 90% of the infections. Approximately half the participants (47%) were prescribed the single tablet formulation of ledipasvir/sofosbuvir.

**Table 1.** Participant Characteristics

	Lottery	Fixed	Overall
Sex, n	31	28	59
Male	23 (74%)	17 (61%)	40 (68%)
Female	8 (26%)	11 (39%)	19 (32%)
Ethnicity, n	31	28	59
Hispanic/Latino	0 (0%)	1 (4%)	1 (2%)
Other	31 (100%)	27 (96%)	58 (98%)
Race, n	31	28	59
White/Caucasian	18 (58%)	11 (39%)	29 (49%)
Black/African American	12 (39%)	13 (46%)	25 (42%)
Indian (American)	0 (0%)	1 (4%)	1 (2%)
Mixed	1 (3%)	2 (7%)	3 (5%)
Latin	0 (0%)	1 (4%)	1 (2%)
Age (years), n	31	28	59
Median (Q1, Q3)	55 (50, 60)	54 (50, 58)	54 (50, 60)
Mean (SD)	55 (9)	53 (10)	54 (9)
Income, n	31	28	59
$0–$20000	24 (77%)	17 (61%)	41 (69%)
$21000–$40000	3 (10%)	5 (18%)	8 (14%)
$41000–$60000	4 (13%)	1 (4%)	5 (8%)
$61000–$80000	0 (0%)	1 (4%)	1 (2%)
Refused	0 (0%)	4 (14%)	4 (7%)
Highest Level of Education, n	30	28	58
No. missing	1	0	1
Middle school (Jr. High) or less	4 (13%)	0 (0%)	4 (7%)
Some high school, no diploma	7 (23%)	5 (18%)	12 (21%)
High school graduate/GED or equivalent	11 (37%)	12 (43%)	23 (40%)
Junior (2 year) college	1 (3%)	1 (4%)	2 (3%)
Technical/trade/vocational school	0 (0%)	3 (11%)	3 (5%)
Some college (4-year college or university)	3 (10%)	5 (18%)	8 (14%)
College graduate (4-year college or university	2 (7%)	1 (4%)	3 (5%)
Postcollege/graduate	1 (3%)	1 (4%)	2 (3%)
Don’t know	1 (3%)	0 (0%)	1 (2%)
Health Insurance Status, n	31	28	59
Insured	25 (81%)	21 (75%)	46 (78%)
Uninsured	6 (19%)	7 (25%)	13 (22%)
Housing Status^a^, n	31	28	59
Stable	18 (58%)	21 (75%)	39 (66%)
Unstable	13 (42%)	7 (25%)	20 (34%)
HCV-RNA (log_10_ IU/mL), n	31	27	58
No. missing	0	1	1
Median (Q1, Q3)	6.27 (5.67, 6.64)	6.06 (5.22, 6.42)	6.12 (5.66, 6.53)
Mean (SD)	6.14 (0.75)	5.95 (0.69)	6.05 (0.72)
Liver Cirrhosis, n	31	28	59
Yes	11 (35%)	13 (46%)	24 (41%)
No	19 (61%)	15 (54%)	34 (58%)
Unknown	1 (3%)	0 (0%)	1 (2%)
HCV Genotype, n	31	28	59
1	26 (84%)	27 (96%)	53 (90%)
2	5 (16%)	1 (4%)	6 (10%)
Documented HIV Status, n	31	28	59
Positive	9 (29%)	10 (36%)	19 (32%)
Negative	11 (35%)	10 (36%)	21 (36%)
Unknown	11 (35%)	8 (29%)	19 (32%)
CES-D Score, n	31	28	59
Median (Q1, Q3)	22 (17, 30)	19 (15, 25)	21 (15, 27)
Mean (SD)	24 (10)	21 (9)	23 (10)
Current Drug-Use Status^b^, n	31	28	59
Active	12 (39%)	15 (54%)	27 (46%)
Not Active	19 (61%)	13 (46%)	32 (54%)
Current Drug(s) Used, n	31	28	59
Marijuana	11 (35%)	10 (36%)	21 (36%)
Hallucinogens	2 (6%)	1 (4%)	3 (5%)
Cocaine/crack	5 (16%)	7 (25%)	12 (20%)
Heroin	2 (6%)	2 (7%)	4 (7%)
Street methadone	0 (0%)	1 (4%)	1 (2%)
Prescription painkillers^c^	1 (3%)	7 (25%)	8 (14%)
Methamphetamines	2 (6%)	0 (0%)	2 (3%)
Stimulants	2 (6%)	1 (4%)	3 (5%)
Tranquilizers/sedatives	1 (3%)	1 (4%)	2 (3%)
Regimen, n	31	28	59
Ledipasvir/sofosbuvir	14 (45%)	14 (50%)	28 (47%)
Ribavirin, ledipasvir/sofosbuvir	1 (3%)	0 (0%)	1 (2%)
Ribavirin, sofosbuvir	5 (16%)	1 (4%)	6 (10%)
Ribavirin, sofosbuvir, interferon	3 (10%)	1 (4%)	4 (7%)
Ribavirin, sofosbuvir, simeprevir	1 (3%)	1 (4%)	2 (3%)
Sofosbuvir, simeprevir	7 (23%)	11 (39%)	18 (31%)

Abbreviations: CES-D, Center for Epidemilogical Studies Depression; GED, General Education Diploma; HCV, hepatitis C virus; HIV, human immunodeficiency virus; RNA, ribonucleic acid; SD, standard deviation.

^a^Housing stability was defined as owning or renting a house or apartment.

^b^Current was defined to be in the past month.

^c^Narcotics available by prescription.

All 31 (100%) of those assigned to the lottery arm and 24 of 28 (86%) assigned to the fixed-incentive arm completed a 12-week course of therapy (Figure 1). Of the 4 who did not, 3 had their treatment duration extended to 24 weeks by the clinician (independent of virologic response or adherence concerns) and 1 was only able to receive 8 weeks of therapy because the payer would not approve the planned final 4 weeks.

After completion of treatment, 2 of 31 participants (6%) in the lottery arm and 1 of 28 (4%) in the fixed-incentive arm were lost to follow-up before ascertainment of SVR. In addition, 1 participant in the lottery arm did not have an SVR measurement (HCV RNA at 8 weeks posttreatment was undetetcable), and in the fixed-incentive group 1 participant died of carcinoma of the lung before the planned SVR visit.

### Financial Incentives

The mean financial incentive per visit provided to participants was $71 (standard deviation [SD] = $18) in the lottery arm and $67 (SD = $9) in the fixed-incentive arm. The mean total financial incentive received was $259 (SD = $74) in the lottery arm and $239 (SD = $58) in the fixed-incentive arm.

### Perceptions Regarding Financial Incentives

Fifteen participants (8 in the fixed-incentive arm and 7 from lottery arm) completed a semistrucutred qualitative interview. Participants expressed general satisfaction with their assigned incentive strategy. The majority of the fixed-incentive arm participants states they preferred the dependability of a predetermined amount and appreciated knowing how much they would receive for completing treatment objectives. Those randomized to the lottery generally found this strategy to be “fun,” and they stated that they liked the chance involved, comparing it to being at “the fair.” When asked which incentive strategy to offer future patients, 75% of the fixed-incentive arm interviewees recommended the fixed-incentive, and 71% of the lottery arm interviewees recommended the lottery.

### Hepatitis C Virus Study Visit and Medication Adherence

Rates of attendance of scheduled clinic visits for HCV care are listed in Table 2. Overall, 92% of scheduled visits were attended (including 95% of the week 12 EOT visits and 88% of SVR visits), and there was no statistically significant difference in missed visits between the study arms. Both the median and mean number of clinic visits attended was 4 in each study arm (for both lottery and fixed incentives; SD = 1).

**Table 2.** Scheduled Visit Attendance

	Lottery	Fixed	Overall	*P* Value
Weed 2 Visit, n	19	10	29	.592
Attended	17 (89%)	8 (80%)	25 (86%)	
Missed Visit	2 (11%)	2 (20%)	4 (14%)	
Week 4 Visit, n	21	23	44	.222
Attended	19 (90%)	23 (100%)	42 (95%)	
Missed Visit	2 (10%)	0 (0%)	2 (5%)	
Week 6 Visit, n	12	6	18	>.999
Attended	12 (100%)	6 (100%)	18 (100%)	
Week 8 Visit, n	15	14	29	>.999
Attended	13 (87%)	13 (93%)	26 (90%)	
Missed Visit	2 (13%)	1 (7%)	3 (10%)	
Week 12 Visit, n	30	27	57	.599
Attended	29 (97%)	25 (93%)	54 (88%)	
Missed Visit	1 (3%)	2 (7%)	3 (5%)	
Week 24 Visit, n (SVR)	31	28	59	>.999
Attended	27 (87%)	25 (89%)	52 (88%)	
Missed Visit	4 (13%)	3 (11%)	7 (12%)	

Abbreviations: SVR, sustained virologic response.

**Figure 2.**
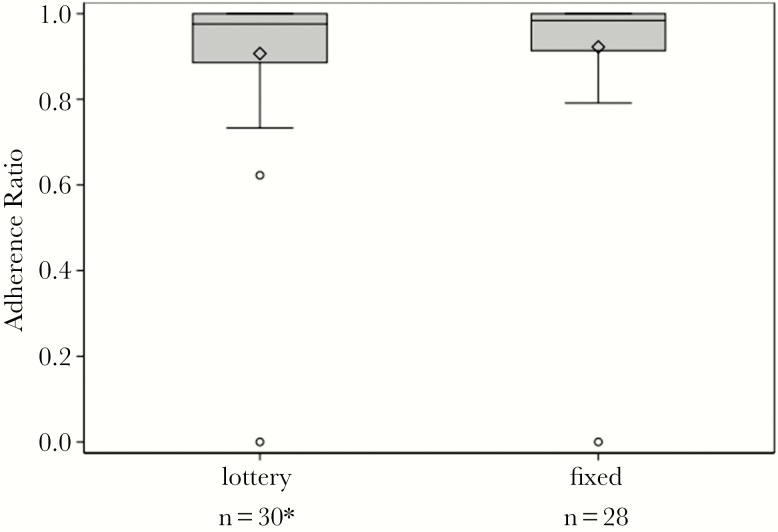
Adjudicated analysis of hepatitis C virus medication adherence (electronic cap). *One lottery arm participant was excluded from the adjudicated electronic medication cap analyses due to inconsistent use of the electronic cap during treatment (participant was also using a pillbox).

Electronic medication monitoring estimated a mean adherence ratio (days with ≥1 bottle opening:monitored days) of 0.91 overall (0.91 for lottery and 0.92 for fixed incentive) in the adjudicated analysis, excluding those without reliable cap data (eg, those who used a pill box) (Figure 2). Adherence of 90% or greater as estimated by electronic monitoring was achieved by 72.4% of particiants including 70% of the lottery and 75% of fixed incentive participants (estimated difference of −5.0%; exact 95% CI, −28.2 to 19.1). Adherence results were very similar in the conservative, unadjudicated analysis (data not shown). Participants brought medication bottles to 85% of scheduled clinic visits for pill counts, and less than 10% had estimated adherence fall below 90% at any monthly visit.

### Rates of Sustained Virologic Response

In the ITT analysis, 92.9% of the lottery arm and 92.3% of the fixed-incentive arm achieved SVR (estimated difference of 0.5%; 95% CI, −17.5 to 18.8). The PP analysis yielded similar results (Table 3).

**Table 3.** Sustained Virologic Response by Study Arm

Endpoint	N	Pooled Proportion (Exact CI)	Lottery	Fixed	Difference (Exact CI)
Intent to treat	54	92.6% (82.1–97.9)	26/28 (92.9%)	24/26 (92.3%)	0.5 (−17.5 to 18.8)
Per-protocol	50	92.0% (80.8–97.8)	26/28 (92.9%)	20/22 (90.9%)	1.9 (−16.0 to 23.8)

Abbreviations: CI, confidence interval.

### Substance Use Screening at Baseline, During and After Hepatitis C Virus Treatment

Fifty-five of 59 participants (93%) had at least 1 urine specimen submitted for toxicology. At baseline, 10 of 48 (21%; 19% of lottery, 23% of fixed) providing urine screened positive for substances of abuse. During the trial, a positive toxicology screen was detected at least once in 31% of the 55 participants providing a urine sample, balanced evenly between the lottery (9 of 29) and fixed-incentive (8 of 26) arms. At the last visit at which a urine specimen was provided, 14 of the 55 (25%) participants tested positive: 28% in the lottery arm and 23% in the fixed-incentive arm.

The estimated relative proportion achieving SVR was not significantly different between those who had and did not have a positive urine test at baseline or the last screen (PR = 1.13; 95% CI, 0.83–1.35). Likewise, the baseline toxicology result was not associated with achieving >90% HCV medication adherence (PR = 1.09; 95% CI, 0.48–1.51).

## DISCUSSION

Financial incentives to encourage participation and adherence in HCV care among patients with a history of substance abuse were found to be feasible in this pragmatic pilot study. More than 90% of treatment-related visits were attended, and there was a low rate of study withdrawal. In addition, we observed high rates of both adherence to HCV DAA medication and care visits, with on average over 90% of prescribed doses of sofosbuvir-containing regimens taken during the 12-week course of therapy, according to electronic medication monitoring. Given this, it is unsurprising that 92% of participants achieved SVR. Most importantly, study participants found the study procedures and both financial incentives acceptable and feasible.

Rates of HCV-related clinical care and medication adherence and SVR achievement were similar with the 2 strategies of financial incentives tested: 1 based on a lottery and the other a fixed schedule of payments. There were no significant differences between the study arms in the proportion who dropped out or were lost to follow-up, suggesting that both strategies were acceptable to patients. Furthermore, under the conditions studied, the costs of each approach were similar.

Because the convenience, tolerability, and potency of treatment for HCV have increased, it can be argued that there is little need to incentivize adherence to HCV medications or clinical supervision. However, as HCV care expands to include those who were previously considered poor candidates for treatment, including those with active substance use and mental health disorders, the extremely high SVR rates seen in clinical trials of these medications may be lower in more real-world conditions among patients faced with barriers to entering and persisting in care. Given the high cost of HCV DAAs, the investment of a fraction of this expense to support treatment adherence and completion for even a small number of patients at risk for virologic failure may be cost-saving. It is notable that during this pilot, half of the participants were actively using substances of abuse at baseline.

Lottery-based incentives have been used to support a number of other health behaviors including smoking cessation, weight loss, cancer screening, and antiretroviral therapy. Moreover, these incentives may be particularly effective for individuals, such as those with serious substance use disorders, who tend toward risk-taking endeavors [29, 33]. In this pilot comparison with predictable financial incentives, the lottery-based strategy seemed to be as acceptable and effective for promoting HCV treatment. Qualitative data collected at the end of the trial found that participants from both study arms had favorable views of the financial incentives as a tool to support HCV treatment, and those in the lottery-based arm found the strategy to be novel and, even, fun.

In preparation for a future randomized controlled trial, the primary foci of this pilot study were to examine the feasibility of delivering financial incentives for HCV patients and patients with substance abuse issues and ensure that the study procedures and incentives were acceptable to patients; therefore, all participants received financial incentives. The influence of financial incentives on the clinical outcomes of interest cannot be determined from this study, because there was no nonincentivized control group. Any future randomized controlled trial should compare one of these financial strategies to standard of care in an appropriately at-risk population to determine the efficacy and effectiveness of this intervention.

In addition to a design that precludes the determination of the effectiveness of financial incentives for HCV care and medication adherence, the interpretation of this study should also take into account that it was conducted at a single academic medical facility in the southeastern United States and may not generalize to other settings and locations. For this preliminary investigation of financially incentivizing HCV care, we included patients with current and previous abuse of a variety of substances. The outcomes we observed may have been different in cohorts with higher rates of active abuse of cocaine, methamphetamines, and/or opiods. Likewise, in this relatively small pilot, opportunities for analyses by subgroups such as gender, ethinicity, and race were not possible. Furthermore, there are well described limitations of both pill count and electronic monitoring for estimating medication adherence [46]. We applied both a conservative (unadjudicated) and more liberal (adjudicated) approach to the analysis of electronic medication adherence data and found the differences between these analyses to be small. Substance use was assessed by self-report, which may suffer from social desirability bias. Finally, although urine toxicology was performed, which is a strength, participants did not always provide a requested urine specimen at each visit and therefore there are missing data; however, 55 of 59 participants provided at least 1 single urine specimen for toxicology testing at some point during the trial.

## CONCLUSIONS

In conclusion, 2 different incentive strategies were successfully implemented with patients with a history of substance use disorders. High levels of adherence to newer DAAs for HCV and to HCV clinical care were observed. These results support the further testing of financial incentives for HCV treatment with a controlled design, especially in patients with substantial challenges to adherence due to substance abuse disorders.
